# Doppler ultrasound evaluation in preeclampsia

**DOI:** 10.1186/1756-0500-6-477

**Published:** 2013-11-19

**Authors:** Maria A Lopez-Mendez, Victoria Martinez-Gaytan, Raul Cortes-Flores, Rene M Ramos-Gonzalez, Mauro A Ochoa-Torres, Idalia Garza-Veloz, Monica I Martinez-Acuña, Jose I Badillo-Almaraz, Margarita L Martinez-Fierro

**Affiliations:** 1 Departamento de Radiologia e Imagen, Unidad Medica de Alta Especialidad No. 25 Instituto Mexicano del Seguro Social, Monterrey, Mexico; 2 Unidad Medica Materno-Fetal del Noreste, Unidad Medica de Alta Especialidad No. 23 Instituto Mexicano del Seguro Social, Monterrey, Mexico; 3 Molecular Medicine Laboratory, Unidad Academica de Medicina Humana y Ciencias de la Salud, Universidad Autonoma de Zacatecas, Carretera Zacatecas-Guadalajara Km. 6, C.P. 98160 Zacatecas, Mexico

**Keywords:** Doppler velocimetry, Preeclampsia, Uterine artery, Umbilical artery, Middle cerebral artery

## Abstract

**Background:**

Worldwide preeclampsia (PE) is the leading cause of maternal death and affects 5 to 8% of pregnant women. PE is characterized by elevated blood pressure and proteinuria. Doppler Ultrasound (US) evaluation has been considered a useful method for prediction of PE; however, there is no complete data about the most frequently altered US parameters in the pathology. The aim of this study was to evaluate the uterine, umbilical, and the middle cerebral arteries using Doppler US parameters [resistance index (RI), pulsatility index (PI), notch (N), systolic peak (SP) and their combinations] in pregnant women, in order to make a global evaluation of hemodynamic repercussion caused by the established PE.

**Results:**

A total of 102 pregnant Mexican women (65 PE women and 37 normotensive women) were recruited in a cases and controls study. Blood velocity waveforms from uterine, umbilical, and middle cerebral arteries, in pregnancies from 24 to 37 weeks of gestation were recorded by trans-abdominal examination with a Toshiba Ultrasound Power Vision 6000 SSA-370A, with a 3.5 MHz convex transducer. Abnormal general Doppler US profile showed a positive association with PE [odds ratio (OR) = 2.93, 95% confidence interval (CI) = 1.2 - 7.3, *P* = 0.021)], and a specificity and predictive positive value of 89.2% and 88.6%, respectively. Other parameters like N presence, RI and PI of umbilical artery, as well as the PI of middle cerebral artery, showed differences between groups (*P* values < 0.05).

**Conclusion:**

General Doppler US result, as well as N from uterine vessel, RI from umbilical artery, and PI from umbilical and middle cerebral arteries in their individual form, may be considered as tools to determine hemodynamic repercussion caused by PE.

## Background

Worldwide pre-eclampsia (PE) is the first cause of maternal mortality, intrauterine growth retardation (IUGR), and fetal prematurity [1,2]. PE affects 5-10% of pregnancies and is clinically manifested after 20 weeks of gestation (GW) [3,4]. The etiology of PE is still unknown, although an excessive maternal systemic inflammatory response and an imbalance between circulating angiogenic and anti-angiogenic factors have been described [5,6]. The pathophysiology of PE is based on the incapability of the trophoblast to invade properly the myometrium causing a limited remodeling of spiral arteries [7]. The impaired placental perfusion caused by vascular abnormalities precedes clinical manifestations of PE and it can be detected by Doppler ultrasound (US). The latter has been considered a useful method for prediction of PE and adverse pregnancy outcome [8,9]. Uterine artery is the most studied vessel in the Doppler evaluation in PE, because it represents the maternal vascular condition, through the pulsatility and resistance index (PI and RI respectively) and the presence of early diastolic notch (N) [10,11]. Although there are some studies including the umbilical artery as a relevant vessel in the PE evaluation, traditionally this artery is taken together with the middle cerebral artery in the fetus status evaluation [12-15]. In PE evaluation using Doppler US, there is no complete data about the most frequently altered US parameters in their individual or combined form for each artery. In this study we evaluate the uterine, umbilical, and the middle cerebral arteries using several Doppler US parameters (RI, PI, N, SP and their combinations) in order to make a global evaluation of hemodynamic repercussion caused by the established PE, in a cases and controls study.

## Methods

### Patients

The patients were recruited from the high risk consult of the Unidad Medica de Alta Especialidad (UMAE) # 23 of the Instituto Mexicano del Seguro Social (IMSS) in Monterrey, Mexico, between September 2009 and June 2010.Women from 15 to 40 years old, with singleton pregnancy between the 24–37 GW were included and divided in cases and controls groups. The cases group consisted by diagnosed PE women according to the guidelines of the International Society for the Study of Hypertension in Pregnancy [16]. Most of the patients were not on any treatment at the time of examination, the patients from the cases group with treatment, had been on it for less than 2 days. Medical therapy of these patients was selected according to the Mexican Technical Guideline for Prevention, Diagnosis, and Management of Preeclampsia-Eclampsia [17]. Normotensive women from the control group did not have any hypo tensor treatment at the moment of the US, nor did they have co-morbidities associated to IUGR. Multiple pregnancies, pregnancies with structural or chromosomal fetus malformations, no feasibility to undergo the Doppler US test (obesity, oligohydramnios, etc.) were excluded from the study. The protocol was approved by the Institutional Review Board (ID number R-2010-1905-17). All patients provided written informed consent for their participation.

### Doppler US evaluation

To determine the Doppler US pattern, only one exam on each patient was carried out at the recruitment time. The Doppler US was performed by trans-abdominal examination with a Toshiba Power Vision 6000 SSA-370A model, with a 3.5 MHz convex transducer. The examination included one uterine artery from the placental side or the mean if there was a symmetrical placenta, the umbilical artery, and one middle cerebral artery (indistinct hemisphere) [18,19]. Umbilical artery measures were taken in a free umbilical cord loop. In order to register the values, four out of five spectral continuous and identical waves were considered, after verification of regular maternal and fetal cardiac frequency, without breath and/or fetal movement interference. The Doppler insonation angle was maintained below 60 degrees. All the US evaluations were performed by a gynecologist with maternal-fetal medicine experience. The results were documented on a record sheet designed for the study, as well as in the clinical record of each patient. The abnormality in the wave morphology was considered as the presence of a protodiastolic N after the 24 GW in the uterine artery [20]. RI and PI values above the 95^th^ percentile standardized for the gestational age were considered abnormal for the uterine and umbilical arteries, and below the 10^th^ percentile for the middle cerebral artery [20,21]. Alterations in any of the uterine artery parameters were interpreted as an abnormal result of this artery and consequently an abnormal general Doppler result was reported. In the umbilical vessel, alterations in the individual or combined RI and PI values were reported as an abnormal artery result. Decreases of individual RI or PI values, as well as their combination in the middle cerebral artery were considered as an abnormal artery result. For umbilical and middle cerebral vessels only their combination with another abnormal artery was considered to report an abnormal general Doppler result.

### Statistical analysis

Chi-square or Fisher’s exact test was used to analyze categorical variables. Unpaired *t*-test and Mann–Whitney Rank Sum test were used for continuous variables; *P* values were corrected by maternal age using a multiple logistic regression analysis. Sensitivity, specificity, predictive positive (PPV) and negative (NPV) values were calculated according to the Bayes theorem. Statistically, *P* values < 0.05 were considered significant. Statistical analysis was performed using the SigmaPlot software v11.

## Results

A total of 102 Mexican women were recruited and sub-divided in two groups: 65 formed the cases group (38 mild and 27 severe PE) and 37 were the control group. 56.9% of the cases were diagnosed with PE before 34 GW (early PE) and the remaining 43.1% had a late onset of disease (34–37 GW). General characteristics of the study population are shown in Table 1. The median of GW was 34 for the cases (ranging from 24.5 to 37 GW) and 32 (ranging from 24 to 37 GW) for controls, respectively. Maternal age, was the only characteristic with statistical difference among groups (29.1 for the cases and 26.1 for the controls, *P* = 0.019). There were no differences in risk factors such as past personal or familial history of PE, primipaternity and nulliparity, among study groups (Table 1).

**Table 1:** Comparison of general characteristics between study groups

**Characteristic**	**PE**	**Controls**	***P *** **value**
	**(n = 65)**	**(n = 37)**	
Maternal age years, mean (range)	29.1 (15–42)	26.1 (17–36)	0.019^*^
Gestational age weeks, median (range)	34 (24.5-37)	32 (24–37)	0.084
Number of pregnancies, median (range)	2 (1–9)	2 (1–6)	0.608
Past history of PE, n (%)	10 (15.4)	1 (2.7)	0.098
Familial history of PE, n (%)	9 (13.8)	1 (2.7)	0.141
Primipaternity n (%)	24 (36.9)	11 (29.7)	0.604
Nulliparous, n (%)	26 (40.0)	11 (29.7)	0.410
Blood pressure systolic, median (range)	140 (130–179)	100 (90–122)	< 0.001
Blood pressure diastolic, median (range)	90 (50–120)	70 (50–80)	< 0.001

^*^Statistical significance.

Table 2 summarizes the ultrasound findings classified by artery and its comparison between cases and controls. The proportion of patients with PE and an abnormal Doppler US for the uterine artery was statistically significant (OR = 2.6, 95% CI = 1.01 – 6.68, *P* = 0.047). The N presence was restricted only for the cases group in a proportion of 20% (OR = 9.0, 95% CI = 1.127 - 71.887, *P* = 0.032). The medians of RI and PI parameters of the uterine artery showed a close gap between the study groups and therefore they were not associated to PE (*P* values > 0.05). The median of umbilical RI and PI were 0.59 and 0.91 for the cases group and 0.51 and 0.78 for the control group, respectively. There was a positive association between individual values of abnormal umbilical RI or PI and PE (OR = 30.63, 95% CI = 1.47 – 639.71, *P* = 0.027, and OR = 10.82, 95% CI = 2.19 – 53.58, *P* = 0.004, respectively); however, considering the general Doppler result for this artery, differences between proportions of abnormal umbilical Doppler US in the study groups were not observed (*P* = 0.107). An abnormal PI was the only middle cerebral artery parameter associated to PE (OR = 0.243, 95% CI = 0.08 – 0.70, *P* = 0.009).

**Table 2:** Classification of Doppler US findings by artery in the study groups

**Artery**	**Ultrasound finding**	**PE**	**Controls**	***P *** **value**^**†**^
		**(n = 65)**	**(n = 37)**	
Uterine	RI, median (range)	0.54 (0.2-1)	0.51 (0.31-0.62)	0.131
	PI, median (range)	0.84 (0.42-1.7)	0.79 (0.44-1.9)	0.510
	Notch proportion (%)	20	0.0	0.032^*^
	Uterine abnormal Doppler US proportion (%)	44.6	21.6	0.047^*^
Umbilical	RI, median (range)	0.59 (0.31-1.1)	0.51 (0.28-0.76)	0.027^*^
	PI, median (range)	0.91 (0.42-1.9)	0.78 (0.35-1.26)	0.004^*^
	Umbilical abnormal Doppler US proportion (%)	49.2	29.7	0.107
Middle cerebral	RI, median (range)	0.76 (0.5-1)	0.83 (0.4-1.3)	0.500
	PI, median (range)	1.4 (0.9-2.66)	1.815 (1.11-2.81)	0.009^*^
	SP, mean (range)	0.462 (0.22-0.80)	0.434 (0.18-0.60)	0.218
	Cerebral abnormal Doppler US proportion (%)	23.1	18.9	0.911
	Proportion of Abnormal Doppler US (%)	47.7	24.3	0.021^*^

^†^*P* value adjusted by maternal age. ^*^Statistical significance.

**Figure 1:**
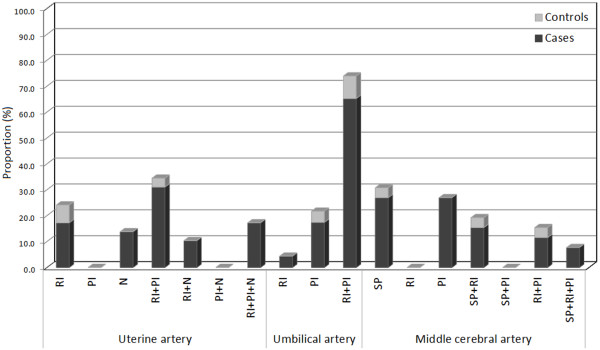
Proportions of the most common abnormal US findings and their combinations for each artery.

Figure 1 shows the frequencies of the most common abnormal US findings, including the parameters combinations for each artery. In the uterine artery, the most frequent abnormal parameter in its individual form and the most frequent abnormal combination for both the cases and controls were the RI (17% for cases and 6.9% for controls) and the RI + PI (31% for cases and 3.4% for controls) respectively. The N presence and its combination with altered RI (RI + N) and the combination between abnormal RI, PI and N (RI + PI + N), only had representation in the cases group. In the umbilical artery the most frequent altered parameter was the RI (17% in the cases group and 4% for the controls). The combination of abnormal RI + PI was detected in 65.2% of the cases with abnormal Doppler US for this artery versus 8.7% of the control pregnancies. Considering the abnormal middle cerebral artery results, 53.8% of women showed altered PI and SP individual parameters in the cases group; this condition was present only in 3.2% of the controls.

Table 3 displays the Doppler US parameters ability to classify the study groups. The general Doppler result had the most representative values with specificity and PPV of 75.7% and 78.6%, respectively. The sensitivity and the NPV for the general US examination were calculated in 50.8% and 46.7%, respectively.

**Table 3:** Effectiveness of Doppler US in PE discrimination: accuracy of general Doppler US result by artery and their combinations

**Artery**	**Parameter**	**Sensitivity (%)**	**Specificity (%)**	**PPV**^**† **^**(%)**	**PNV**^**‡ **^**(%)**
Uterine	RI	7.7	94.6	71.4	36.8
	N	6.2	100.0	100.0	37.8
	RI + PI	13.8	97.3	90.0	39.1
	RI + N	4.6	100.0	100.0	37.4
	RI + PI + N	7.7	100.0	100.0	38.1
	Abnormal Doppler US of uterine artery	44.6	78.4	78.4	44.6
Umbilical	RI	1.5	100.0	100.0	36.6
	PI	6.2	97.3	80.0	37.1
	RI + PI	23.1	94.6	88.2	41.2
	Abnormal Doppler US of umbilical artery	49.2	70.3	74.4	44.1
Middle Cerebral	RI	10.8	100.0	100.0	38.9
	SP	10.8	97.3	87.5	38.3
	PI + SP	6.2	97.3	80.0	37.1
	RI + PI	1.5	91.9	25.0	34.7
	SP + RI + PI	3.1	100.0	100.0	37.0
	Abnormal Doppler US of cerebral artery	23.1	81.1	68.2	37.5
	Abnormal Doppler US	50.8	75.7	78.6	46.7

^†^PPV: Predictive positive value. ^‡^PNV: Predictive negative value.

## Discussion

Most of reports related to PE evaluation by Doppler US have been focused on the study of the uterine artery and its parameters [22-24]; there are descriptive trials about the hemodynamic changes during pregnancy [25], studies to determine the normality ranges of the Doppler US values in some populations [26,27], and reports in which the aim has been to get a spectral pattern of Doppler US to predict which pregnancies will evolve to PE [28-31]. Only a few papers provide an extended description of other vessels in PE evaluation, and even fewer have shown the combination of two or more arteries with the objective of establishing a more informative and accurate report [32-35]. In this study, we report a full Doppler US vision about PE induced vascular changes in the mother, reflected as vascular changes in the uterine artery, and in the fetus, considered as alterations in umbilical and middle cerebral artery parameters; additionally, these abnormal Doppler US measurements were disaggregated for each examined vessel.

In our study, maternal age was the only known PE risk factor with differences among groups [36,37]. Despite the fact that nulliparity, personal history and/or family of PE have been supported as PE risk factors in several studies [38,39], our results showed no association between these characteristics and PE; however, these data should be cautiously interpreted due to the small number of patients included in the study.

Considering the Doppler US findings for motherhood status, from individual parameters evaluated in the uterine artery, N showed to be the most meaningful (but not the most frequent) individual finding, due to the fact that its presence was restricted to the cases group. These results are similar to those reported previously, where the N is considered as a relevant parameter in the uterine artery study [40,41]. Although PI and RI are considered fundamental values in the uterine artery evaluation, we did not find differences in these parameters among the study groups. It could be because the reference values considered in previous studies consisted of standardized measures for each population, and the abnormality limits of these values may differ between populations [40].

In the fetal condition, the umbilical artery has been related typically to pregnancy outcome, while the middle cerebral artery has been considered as a fetal circulation marker and a useful non-invasive tool in risk assessment for fetal anemia [42-45]. In our study, as individual measurements, abnormal RI from umbilical artery, and altered PI from umbilical and middle cerebral arteries, were associated to PE; however, considering all the parameters reviewed in these arteries, the general results for each vessel did not show a difference between groups, suggesting that these individual parameters, but not the general Doppler US for these arteries, could be considered as indicators to evaluate the specific PE vascular alterations.

Even though PE prediction was not the aim of this study, the capability of Doppler assessment to classify correctly the study groups was determined using individual parameters and their combinations. In general, individual or combined measurements showed high specificity and PPV, conversely to the low sensitivity and NPV. Our findings reflected a wide gradient in the specificity/sensitivity results with a screening parameter-dependent variation that may explain the conflicting results in the diagnostic accuracy of Doppler flow velocity to predicting PE in the previous reports. On the other hand, these results provide new information about the relevant US indicators for the pathology and it could contribute to generate more descriptive and accurate reports during the PE evaluation using Doppler assessment. In Mexico, there are no reports about the Doppler US features for the three arteries in the PE evaluation; our results provide evidence about the possibilities to select better and specific measurements of each vessel in future early PE Doppler US screenings.

## Conclusion

General Doppler US result, as well as N from uterine vessel, RI from umbilical artery, and PI from umbilical and middle cerebral arteries in their individual form, may be considered as tools to determine hemodynamic repercussion caused by PE.

## Abbreviations

PE: Preeclampsia; US: Ultrasound; RI: Resistance index; PI: Pulsatility index; N: Notch; SP: Systolic peak; IUGR: Intrauterine growth retardation; GW: Weeks of gestation; PPV: Predictive positive value; NPV: Negative predictive value.

## Competing interests

The authors declare that they have no competing interests. The authors are responsible for the content and writing of the research paper.

## Authors’ contributions

MALM, MLMF, drafted the manuscript. MALM, VMG, RCF, RMRG, MAOT, and MLMF, participated in the design of the study and in the coordination of the patients’ recruitment. MALM, VMG, RCF, RMRG, MAOT, IGV, MIMA, and JIBA, participated in data acquisition, data analysis and interpretation, they participated in manuscript review. MLMF conceived of the study, and participated in its design and coordination, and performed the statistical analysis. All authors read and approved the final manuscript.
